# Bipolar Saline TURP for Large Prostate Glands

**DOI:** 10.1100/tsw.2007.241

**Published:** 2007-09-17

**Authors:** David S. Finley, Shawn Beck, Richard J. Szabo

**Affiliations:** ^1^ Department of Urology, University of California Irvine Medical Center, Orange, CA, USA; ^2^ Department of Urology, Kaiser Permanente Medical Center, Irvine, CA, USA

**Keywords:** adenoma, prostate, TURP, bipolar, saline

## Abstract

The objective of this study was to evaluate the feasibility of bipolar transurethral resection of the prostate (TURP) in patients with very large prostate glands and significant comorbidities. Four patients with prostate glands >160 cc on preoperative volume measurement and ASA class three or higher underwent bipolar TURP with the Gyrus PlasmaKinetic system. Preoperative, operative, and postoperative parameters were studied. The results showed an average ASA class 3.25 (range: 3–4). The average preoperative prostate volume was 207.4 cc (range: 163–268). The average preoperative International Prostate Symptom Score (IPSS) and bother score was 31 and 6, respectively. Mean resection time was 163 min (range: 129–215). The weight of resected tissue and percentage of vaporized tissue was 80.8 g (range: 62–115) and 10.0% (range: 3.8–15.1), respectively. An average of 61L of saline was used (range: 48–78). The mean change in hemoglobin and serum sodium was 2.1 g/dl (range: 1.4–2.7) and 3.3 meq/l (range: 2–4), respectively. Postoperative catheter time averaged 76 h (range: 40–104). Mean length of hospital stay was 12 h (range: 4–24). The mean postoperative IPSS and bother score was 2.75 and 0.25, respectively. Bipolar TURP is a feasible alternative to simple open prostatectomy in high-risk patients with massive prostate adenomas. Prostate volume is reduced by approximately 10% due to vaporization.

## INTRODUCTION

Monopolar transurethral resection of the prostate (TURP) remains the gold standard for the surgical treatment of benign prostatic hyperplasia (BPH). Most surgeons feel comfortable resecting moderate- to large-sized glands (40–60 cc) using monopolar systems. Concerns over dilutional hyponatremia and TUR syndrome with the use of hypotonic irrigant, however, generally limit safe resection times.

The management of very large prostate glands (i.e., >100 cc) poses a special challenge. Various techniques have been used, including simple prostatectomy (open, laparoscopic, robotic), monopolar TURP, staged TURP (hemiresection), Holmium laser enucleation of the prostate (HoLEP), Nd:Yag laser ablation (VLAP), and bipolar saline TURP.

The bipolar resectoscope has become increasingly popular because it utilizes saline irrigant, which avoids the potential for dilutional hyponatremia and the associated constraints on resection time. With a rapidly growing elderly population, the number of high-risk patients with very large prostate glands is expanding. Many of these patients would be unable to tolerate the morbidity associated with radical surgery and general anesthesia.

We present our initial experience with bipolar saline TURP under spinal anesthesia in medically compromised patients with massive prostate glands.

## PATIENTS AND METHODS

We evaluated the efficacy of saline TURP for prostate glands measuring 160–268 cc utilizing the Gyrus bipolar system in four high-risk patients. All patients underwent surgery under spinal anesthesia at Kaiser Permanente Medical Center in Irvine, CA during a 2-month period. All patients were considered high operative risk (ASA class ≥3) for simple open prostatectomy. Patients were counseled on the risks, benefits, and alternatives of surgery and provided informed consent. All patients were cleared for spinal anesthesia by anesthesiology after medical and/or cardiology clearance.

All patients were diagnosed with symptomatic BPH according to International Prostate Symptom Score (IPSS), bother score, uroflowmetry, postvoid residual volume (PVR), digital rectal exam, quantification of prostate volume by transrectal ultrasound (n = 3), or CT scan (n = 1). Whole prostate volume was calculated according to the formula: (length × width × height × π/6).

All patients underwent a bipolar saline TURP utilizing the Gyrus PlasmaKinetic System (Gyrus Medical System, Minneapolis, MN) with a 26Fr continuous flow resectoscope. A superloop was utilized on the following settings: Thermosect (T2) or PS2 at 160 W and Dessicate at 120 W. A 24Fr two-way Foley catheter was placed postoperatively and hand irrigated as needed. Continuous bladder irrigation was not used.

A postoperative transrectal ultrasound was performed on all patients several weeks postoperatively. The percentage of vaporized tissue was calculated as follows: ([preoperative prostate volume {g} – resected weight {g} – postoperative prostate volume {g}/preoperative volume {g}]) × 100.

Patients were seen postoperatively at 1 week, 3 months, and 6 months. Postoperative IPPS and bother score were elicited at 6 months.

## RESULTS

The indications for surgery included urinary retention and severe lower urinary tract symptoms (LUTS) in three patients, two of whom had a chronic indwelling catheter and one patient with recurrent prostatic bleeding with severe LUTS. Malignancy was excluded by PSA and prostate biopsy when indicated. Two patients were on finasteride preoperatively. All patients failed alpha-blocker therapy. The average preoperative IPSS and bother score was 31 (severely symptomatic) and 6 (terrible), respectively.

The average patient age was 69.3 years (range: 61–76) and ASA class 3.25 (range: 3–4). The average calculated preoperative prostate volume was 207.4 cc (range: 163–268) (Table 1). The mean resection time was 163 min (range: 129–215) (Table 2). The average weight of resected tissue was 80.8 g (range: 62–115). The mean percentage of vaporized tissue was 10.0% (range: 3.8–15.1, SD = 5.8). An average of 61 l of saline were used (range: 48–78) (Table 2). The mean change in hemoglobin and serum sodium was 2.1 g/dl (range: 1.4–2.7) and 3.3 meq/l (range: 2–4), respectively. The average postoperative catheter time was 76 h (range: 40–104). The mean length of hospital stay was 12 h (range: 4–24).

Intraoperatively, a strong obturator reflex was observed several times in two cases during lateral lobe takedown near the bladder neck. No patient required blood transfusion. All patients voided spontaneously after catheter removal. Mean change in PVR was 608 cc (range: 469–800). There were no immediate or early complications. At 6-month follow-up, there were no complications.

The average postoperative IPSS and bother score was 2.75 (mildly symptomatic) and 0.25 (delighted), ($p < 0.001$).

**TABLE 1**
**Patient Characteristics**

| Patient | Age | ASA | Indication | TRUS Vol | Pre-PVR | Pre-IPSS and Bother |
|---|---|---|---|---|---|---|
| 1 | 73 | 3 | BOO | 163 | 728 | 34, 6 |
| 2 | 76 | 3 | Hematuria/LUTS | 179 | 0 | 25, 6 |
| 3 | 67 | 4 | BOO | 171 | 519 | 33, 6 |
| 4 | 61 | 3 | BOO | 268 | 800 | 32, 6 |

ASA, American Society of Anesthesiologists score; TRUS Vol, transrectal ultrasound volume; BOO, bladder outlet obstruction; PVR, postvoid residual; IPSS, International Prostate Symptom Score; LUTS, lower urinary tract symptoms.

**TABLE 2**
**Outcomes**

| Patient | OR Time (min) | LOS (h) | ΔHgb (g/dl) | ΔNa (meq/l) | Catheter (h) | Path (g) | Post-PVR (ml) | *Post-IPSS, Bother |
|---|---|---|---|---|---|---|---|---|
| 1 | 129 | 0 | 2.4 | 2 | 104 | 72 | 0 | 3, 0 |
| 2 | 215 | 24 | 1.4 | 4 | 40 | 115 | 0 | 1, 0 |
| 3 | 168 | 24 | 2.7 | 4 | 96 | 74 | 50 | 4, 0 |
| 4 | 140 | 0 | 1.7 | 3 | 72 | 90 | 0 | 3, 1 |

LOS, length of stay; Na, serum sodium; Path, pathologic weight of resected chips; PVR, postvoid residual; IPSS, International Prostate Symptom Score.

* 6-month postop.

## DISCUSSION

The surgical options for the treatment of very large prostate glands (i.e., >80 g) historically have been limited to open prostatectomy or by performing hemiresection across multiple sessions. Mebust et al., in the AUA cooperative study, reported that the risk of complications with single-session TURP increases significantly with glands >45 g and resection times longer than 90 min[1]. Agarwal et al. found that morbidity and mortality rates were directly proportional to resection weight with weights of 80 and 100 g carrying morbidity and mortality rates of 55 and 6%, and 88 and 22%, respectively[2].

Recently, several endoscopic modalities have been developed to challenge this notion. Both holmium and Nd:YAG lasers have been used for the treatment of large prostate glands. Holmium laser enucleation of the prostate (HoLEP) compared favorably against simple open prostatectomy in a retrospective series of 20 patients with prostate volumes >100 g[3]. In a prospective study of HoLEP in which patients were stratified according to prostate volume, there was no difference in complication rate, catheter time, or outcomes for patients with glands >80 g[4]. HoLEP, however, has a considerable learning curve and can be cumbersome, requiring the use of monopolar electrocautery or a mechanical morcellator to remove the tissue.

Bipolar technology has emerged as another alternative to standard monopolar TURP for the treatment of large adenomas. A small number of studies have demonstrated its feasibility for this application. Botto et al. performed bipolar TURP in 42 patients utilizing a 27Fr sheath; nine patients had glands >60g[5]. The average drop in hemoglobin was 0.43 g/dl. Peak flow rates and IPSS scores at 3 months improved by

140 and 45%, respectively; 4.8% of patients developed urethral stricture. Issa et al.[6] retrospectively reviewed the data from a subset of bipolar TURPs with large resection weights (preoperative prostate volume was not determined). The average resection weight in their series of five patients was 49.6 g over an operative time of 2 h and 22 min, with no reported complications. The mean change in serum sodium and hematocrit was 1.6 mg/dl and 5.6%.

Does the endoscopic treatment of large prostate glands result in increased morbidity (i.e., urethral stricture rate, blood loss)? Head-to-head prospective randomized trials of bipolar vs. monopolar TURP for average-size glands have shown comparable rates of urethral stricture and transfusion[7,8,9,10]. The rate of urethral stricture in the literature for monopolar TURP ranges from about 2–10% over an average resection time of 49 min[11]. Several prospective comparisons of bipolar to monopolar TURP utilizing 26Fr-27Fr resectoscope sheaths have reported urethral stricture rates of 0–4.1% in the bipolar group vs. 1.3–1.9% in the monopolar group for 40- to 50-g prostates after average resection times of 52 and 51 min, respectively[12,13]. Whether or not longer operative times during large adenoma resection are associated with an increased rate of urethral stricture rate remains to be reported.

Although the use of saline irrigation results in minimal sodium shifts, large amounts of isotonic fluid absorption can still result in vascular overload and pulmonary edema[14]. Akcayoz et al. compared fluid absorption during bipolar and monopolar TURP by using irrigant laced with ethanol and then measuring the concentration of the alcohol in ventilated air[15]. After 30 min of resection, bipolar TURP resulted in half the fluid absorption compared to monopolar TURP (689 vs. 1299 ml). We utilized an average of 61 l of saline during our cases, but did not observe any adverse effects secondary to fluid absorption.

Destruction of prostate tissue by bipolar electrovaporization occurs by a combination of electrosurgical vaporization and dessication[16]. The fraction of the total resected weight that occurs due to vaporization is undefined. We performed prostate volume measurements before and after TURP to estimate the amount of tissue that is vaporized during bipolar resection. We subtracted the resected tissue weight from the difference between the calculated pre- and postoperative prostate volumes. We found vaporization accounted for approximately 10% of the total resected weight (range: 3.7–15.1, SD = 5.7). This finding has not been previously reported.

Bipolar electrovaporization causes less heat damage to surrounding tissue. The mean depth of the thermal artifact zone was 0.237 mm (range: 0.060–0.469) in the bipolar group compared with 0.260 mm (range: 0.080–0.410) in the monopolar group[17,18]. As a corollary to this effect, Shiozawa et al. found bipolar transurethral resection in pigs inhibited the obturator nerve reflex[19]. They used a small loop with the TURIS system at 280 W cutting and 120 W coagulation. Similarly, Valdivia et al. noted that bipolar electrosurgery did not cause electromuscular stimulation[20]. In our series of large prostate glands, however, we observed a very strong obturator reflex in half of our cases, an effect typically only seen during lateral wall bladder tumor resection. It is possible that obturator stimulation resulted from near physical contact between the massive lateral lobes and the obturator nerves. Alternatively, this may have been due to the particular combination of thermosect and dessicate settings that we used. Simply lowering the power settings may help to prevent an obturator reflex. Converting from a regional to general anesthetic with neuromuscular blockade would also eliminate this problem.

Bipolar technology allows older, high-risk patients to undergo minimally invasive surgical treatment for BPH under spinal anesthesia. The average ASA score in our series was 3.25. These patients most likely would not have been able to tolerate general anesthesia or the morbidity associated with open surgery. Our average change in hemoglobin and sodium was 2.1 g/dl and 3.3 meq/l, respectively. Patients were sent home the day of the operation or the following morning. In this pilot series, no transfusions or complications have been noted. In addition, we observed an excellent clinic response, with a dramatic change in IPSS and bother scores.

In our initial clinical experience, we were able to safely perform transurethral prostatectomy with the Gyrus bipolar system without complications. Although the resection of very large glands is technically feasible, it is clear that a very skilled resectionist is still required, even with the luxury of saline irrigation.
